# Dietary Supplementation of Astragalus Polysaccharides Enhanced Immune Components and Growth Factors EGF and IGF-1 in Sow Colostrum

**DOI:** 10.1155/2017/9253208

**Published:** 2017-01-09

**Authors:** Lunbo Tan, Ting Wei, Anwen Yuan, Jun He, Jinhui Liu, Daojun Xu, Qing Yang

**Affiliations:** ^1^College of Veterinary Medicine, Hunan Agricultural University, Changsha 410128, China; ^2^College of Animal Science and Technology, Hunan Agricultural University, Changsha 410128, China

## Abstract

Colostrum is the main external resource providing piglets with nutrients and maternal immune molecules. Astragalus polysaccharides (APS) have been used as immunopotentiators in vitro and several animal models. This study aimed to determine the effects of APS on immune factors in sow colostrum and milk. The sow diet was supplemented with APS one week before the expected delivery date. Colostrum and milk were collected and designated as 0 h- (onset of parturition), 12 h-, and 24 h-colostrum and 36 h-milk postpartum. Samples were measured using porcine immunoglobulin (Ig) G, IgM, classical swine fever virus antibody (CSFV Ab), epidermal growth factor (EGF), and insulin-like growth factor- (IGF-) 1 ELISA Quantitation Kits. Dietary supplementation of APS significantly enhanced the presence of IgG, IgM, EGF, and IGF-1 in 0 h-colostrum (*P* < 0.001). The blocking rates of CSFV Ab were increased in samples from APS-supplemented sow when compared to those from the matched samples without APS treatment. The results indicate that supplement of APS could improve the immune components in sow colostrum and/or milk; and status of some specific vaccination could be determined through using colostrum or early milk in sow.

## 1. Introduction

Newborn piglets can hardly obtain passive immunity from maternal blood during fetal period because of the special epitheliochorial structure of pig placenta. Before their own immune system is fully developed, colostrum is the sole external resource which provides piglets with nutrients, maternal immune molecules, and growth factors [1, 2]. Colostrum production lasts for 24 h after the onset of parturition in swine; afterwards, breast secretion is called milk [3]. The maternal molecules include nonspecific immunoglobulins like immunoglobulin (Ig) G, IgM, and IgA as well some specific antibodies [4, 5]. Maternal blood antibodies in colostrum are transferred to newborn piglets to supply protection against foreign antigens. Piglets have the best maternal immunoglobulin absorption from 4 h to 24 h postpartum, and during this period IgG and IgM are principal immunoglobulins in colostrum; after three days delivery, IgA is the primary immunoglobulin in milk [4, 6]. Factors in colostrum play important roles in promoting the development of the gastrointestinal tract of piglets [5]. Studies indicate that the volume of colostrum intake by piglets is highly related to their health and growth [7, 8].

Astragalus polysaccharides (APS) isolated from a traditional Chinese medicinal herb* Astragalus mongholicus* are potentially used as immunopotentiators which could increase serum antibody titer and enhance secretion of a wide range of cytokines [9–13]. Supplementation of APS could increase the immunostimulatory effects against several animal viruses like H9N2 avian influenza virus, foot and mouth disease virus, Newcastle disease virus, and infectious bursal disease virus [9, 13, 14]. Diarrhea and dyspepsia are common diseases for piglets due to the immature digestive system. Studies indicated that growth factors epidermal growth factor (EGF) and insulin-like growth factor-1 (IGF-1) in colostrum and milk play important roles in piglet intestinal growth and development [15]. In weaned pigs, optimal dietary APS has beneficial effect on piglet growth performance and immune function [16].

To study the effects of APS on immune function in sow colostrum, dietary APS supplementation was administrated prior to one week of parturition; concentrations of nonspecific immune factors IgG and IgM were measured as well as titer of the specific antibody against the classical swine fever virus (CSFV) after vaccination. Levels of growth factors including EGF and IGF-1 were also quantified.

## 2. Materials and Methods

### 2.1. Animals

Twenty crossbred sows (large white × landrace) with same number of parturitions were used from a commercial herd, Tianzhao Garden Animal Husbandry Co. Ltd. (Yueyang City, Hunan Province, China). All sows were vaccinated with a swine fever vaccine (Qianyuanhao Biology Co., Ltd., Beijing, China) on day 25 following the previous parturition. One week prior to the expected date of delivery, all pregnant sows were transferred to individual farrowing crates and randomly separated into two groups as the control group (*n* = 10) and APS group (*n* = 10). The control group was fed a common control diet (Table 1). The APS group received the same diet supplemented with APS powder (1.5 g/day/sow, Beijing Centre Biology Co., Ltd., Beijing, China). All sows were fed two times per day at 09:00 a.m. and 6:00 p.m. and all diets were consumed completely by all sows. After delivery, APS was withdrawn and all sows were fed the same diet. The dose of APS feed was determined according to our pilot trial results (unpublished data). All animal procedures were approved by the Ethical Committee of Hunan Agricultural University.

### 2.2. Sample Collection

Colostrum (3-4 mL per sample) was collected from the first teat of each sow at onset of parturition as 0 h-colostrum. Samples were also collected from the first teat at different time points postpartum (as 12 h- and 24 h-colostrum and 36 h-milk) by using individual artificial milking equipment. Sample collection from the fixed first teat was to avoid the value deviation caused by the location of teat as referred by others [17]. All samples were immediately frozen at −20°C before processing.

### 2.3. Quantitation of IgG and IgM

All samples were centrifuged at 5,000 gravity (*g*) for 30 min at 4°C, and supernatants were collected. IgG and IgM were measured using porcine IgG and IgM ELISA Quantitation Kits (Elabscience Biotechnology Co., Ltd., Wuhan, China) according to the manufacturer's instruction.

### 2.4. Determination of CSFV Antibody

Classical Swine Fever Virus antibodies (CSFV Ab) were determined using a commercial ELISA Quantitation Kit (IDEXX Laboratories, Inc., One IDEXX Drive, Westbrook, Maine, USA). The assay is a blocking ELISA using a microplate coated with CSFV antigen. Each sample was analyzed in duplicate, and the mean OD value of each tested sample (OD_TEST_) and that of the negative control (OD_NEG_) were calculated. The inhibition percentage of each sample was calculated according to the following formula: Blocking% = [(OD_NEG_ − OD_TEST_)/OD_NEG_]*∗*100. The test sample was determined as positive with a blocking rate ≥40% (antibodies are present), or negative with a blocking rate ≤30% (antibodies are absent). Blocking rate was calculated according to the instructions as described by others [18].

### 2.5. Growth Factors EGF and IGF-1 Assay

Growth factors EGF and IGF-1 were assayed in colostrum and milk. Supernatants were diluted in 10-fold (for EGF assay) and 40-fold (for IGF-1 assay), respectively. The different dilutions of these growth factors were chosen according to our pilot trial results. The concentrations of the growth factors were quantified using swine EGF or IGF-1 ELISA Quantitation Kits (Elabscience Biotechnology Co., Ltd., Wuhan, China) according to the manufacturer's instructions.

### 2.6. Statistical Analyses

SAS version 8 was used for the statistical analysis and the test of significance was calculated using two-way ANOVA analysis with Duncan's multiple-range test. Results were presented as mean ± standard deviation (mean value ± SD). Difference was considered significant at a level of *P* < 0.05.

## 3. Results

**Figure 1:**
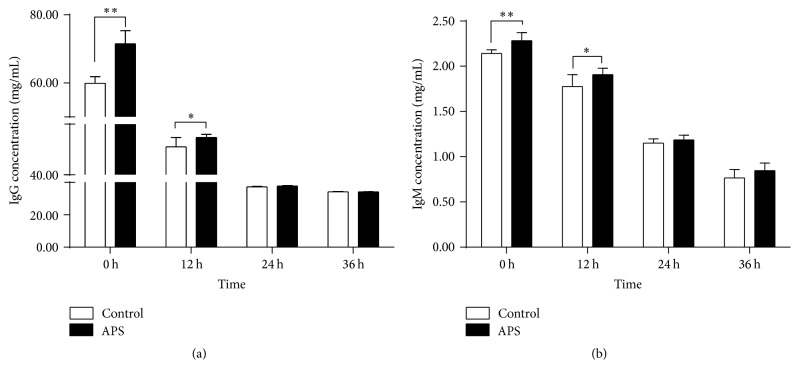
Effects of dietary APS on concentrations of IgG and IgM in sow colostrum. Concentrations of IgG (a) and IgM (b) were measured by ELISA assay. Values are expressed as mean with SD (*n* = 10 for each group). *P* values from two-way ANOVA analysis with Duncan's multiple-range test. ^*∗*^*P* < 0.05 and ^*∗∗*^*P* < 0.01.

As shown in Figure 1, prior to one week of the expected parturition, dietary supplementation of APS significantly enhanced the presence of IgG (Figure 1(a)) and IgM (Figure 1(b)) in 0 h-colostrum (*P* < 0.001) at the onset farrowing and decreased with the time development after delivery. Concentrations of IgG and IgM in 12 h-colostrum were kept higher in APS group when compared to the control group (*P* < 0.05). Twenty-four hours postpartum, both IgG and IgM in colostrum showed no obvious difference between the APS and control groups.

In the present study, blocking rates against CSFV Ab were also determined. As shown in Table 2, CSFV antibody blocking rates from all test colostrum were above 30% with antibodies presence in 0 h- and 12 h-colostrum (blocking rates above 40%), which indicated that vaccination with a swine fever vaccine could obtain good immunity. In APS treated or control group, the blocking rates of CSFV Ab were decreased with the time development. With the supplementation of APS, blocking rates were increased in all indicated time points' colostrum and milk when compared to the controls, and significant differences were observed as *P* < 0.01 in 0 h-colostrum and as *P* < 0.05 in other two time points' colostrum and milk postpartum, respectively.

**Figure 2:**
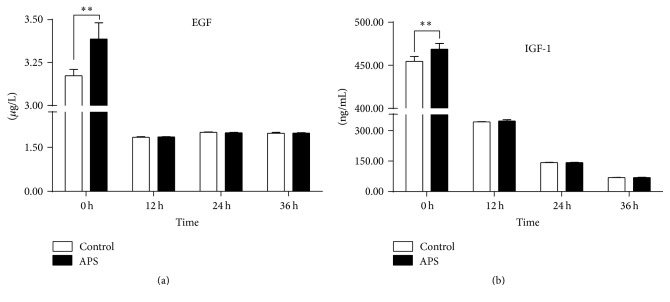
Effects of supplemental dietary APS on concentrations of growth factor EGF (a) and IGF-I (b) in sow colostrum or milk. Growth factors were measured by ELISA assay. Values are expressed as mean with SD (*n* = 10 for each group). *P* values from two-way ANOVA analysis with Duncan's multiple-range test. ^*∗∗*^Values differ significantly between groups at the same indicated time point (^*∗∗*^*P* < 0.01).

Growth factor concentrations of EGF and IGF-1 were measured as shown in Figure 2. Concentrations of EGF were rapidly decreased in colostrum postpartum when compared to the 0 h-colostrum (*P* < 0.001), and dietary supplementation with APS increased the EGF production in 0 h-colostrum (*P* < 0.001) (Figure 2(a)). After farrowing, no difference in EGF was observed between colostrum collected on the indicated time points (Figure 2(a)). The growth factor IGF-1 showed a similar change as EGF that APS increased its production in 0 h-colostrum (*P* < 0.001), but it continued to decrease with the time development postpartum (Figure 2(b)).

## 4. Discussion

In the current field study, supplementation of APS in sow diet markedly improved the levels of IgM and IgG in colostrum one week before the expected delivery date. APS is used to be a immunomodulatory agent to improve growth and immune function in chickens and weaned pigs. Additives of APS in drinking water or diet could increase the average daily gain of chickens and weaned pigs [16, 19]. APS enhanced the production of serum IgM and IgG in aged mice and synergistically resisted the immunosuppression with epimedium polysaccharide in chickens, which are the major immunoglobulins products of humoral immune responses [20, 21]. Therefore, the increase of IgG and IgM upon the APS supplementation in the present study might result from the improvement of humoral immune in the current study.

Vaccination with appropriate vaccines is the main way to prevent the viral infectious diseases. Combined application of a vaccine with an adjuvant or immunomodulator could improve the efficacy of a vaccine. Classical swine fever (CSF) is a contagious viral disease, and mandatory vaccination against CSF is carried out in several countries worldwide. Studies showed that the presence of maternally derived antibodies can protect piglets against this disease [22, 23]. An in vitro study indicated that APS had immunomodulatory effects on peripheral blood mononuclear cells (PBMCs) exposed to CSFV [24]. In the present study, daily supplementation of APS before farrowing could increase the maternally derived specific antibody against CSFV in colostrum which suggested that APS might serve as an effective agent of vaccines against CSFV. Studies showed that APS has beneficial effects on immune response, improving the function of T-lymphocytes and increasing the proliferation of lymphocyte [24–26]. In the present study, the enhancement of CSFV antibody with the dietary APS supplementation in sow could be a result of the increase of the cellular immunity in animals.

Usually, blood is collected from the jugular vein to determine the status of some specific vaccination in sow, which can cause stress and experienced staff is needed in the process of sample collection. Due to relatively high concentrations of immunoglobulins in colostrum, the correlation of maternal* E. rhusiopathiae*- and* M*.* hyopneumoniae*-specific antibody ELISA levels was investigated; the results showed that colostrum has the potential to improve the sensitivity of both ELISAs when compared with serum [27]. In the present study, high levels of specific CSFV antibody were also measured in colostrum, which indicates that colostrum could be used a resource to determine the status of sow immunity.

In colostrum, high content of bioactive compounds such as growth factors, antioxidants, and antimicrobial and immune-modulatory factors can support intestinal maturation, balance and priming of the immune system and establishment of a beneficial gut microbiota [28, 29]. Bovine colostrum can restore intestinal function after initial formula-induced inflammation in preterm pigs [29]. EGF is one of the most abundant growth factors found in the milk and plays important roles in neonatal intestinal development to protect neonates against pathogens infection [30–32]. Plasma anabolic hormones, such as insulin and IGF-1, are highly associated with the local bovine mammary metabolism; highest levels of IGF-1 in milk were found 2 weeks antepartum followed by a rapid decrease during the first milking postpartum [33]. Studies indicated that neonatal tissue development may be improved when piglets consume colostrum with high growth factors such as IGF-1 [34, 35]. In the present study, growth factors EGF and IGF-1 were increased in sow colostrum upon supplementation of APS in diets which may promote the neonatal intestinal development.

In conclusion, APS can improve the concentrations of IgG, IgM, EGF, and IGF-1 in sow colostrum as well the levels of specific antibody against CFSV. Further studies need to be done to investigate the effects of APS on the development and maturation of the newborn piglet intestine.

## Figures and Tables

**(a):** 

Corn	Soybean meal	Rapeseed meal	Barley bran	Calcium dihydrogen phosphate	Calcium carbonate	Compound premix	Salt
65%	15%	10%	5%	0.8%	1%	1.7%	0.5%

**(b):** 

Nutrient components					
ME (MJ/kg)	CP (%)	Ca (%)	P (%)	Lys (%)	Met (%)
13.07	12.943	0.6725	0.594	0.706	0.256

**Table 2:** Effect of APS on blocking rates of CSFV Ab in sow colostrum.

Colostrum	Groups	
	Control	APS
0 h	48.7 ± 4.5^a^	56.4 ± 4.2^a,*∗∗*^
12 h	46.7 ± 3.6^a^	51.3 ± 3.6^b,*∗*^
24 h	38.0 ± 2.8^b^	45.0 ± 5.4^c,*∗*^
36 h	36.2 ± 4.6^b^	43.0 ± 5.5^c,*∗*^

Values (blocking rate, %) are expressed as mean with SD (*n* = 10 for each group). ^a,b,c^Values in the same column differ significantly (*P* < 0.05). ^*∗*,*∗∗*^Values in the same row differ significantly (^*∗*^*P* < 0.05 and ^*∗∗*^*P* < 0.01). *P* values were created from two-way ANOVA analysis with Duncan's multiple-range test.
